# 

**Published:** 1906-07-14

**Authors:** 


### BOOKS RECEIVED.

The Scientific Press, Ltd.

"Muscles and Nerves." By Louis B. Rawling, F.R.C.S.

W. B. Clive.

"Matriculation Directory." University Tutorial Series. No. XLIII. June, 1906.

### MEDICAL VACANCIES.

Bradford Royal Infirmary.—Dispensary Surgeon.
Burnley, Victoria Hospital.—Resident Medical Officer.
Cancer Hospital, Fulham Road, London, S.W.—Medical Officer in Charge of Electrical Department.
Central London Throat and Ear Hospital, Gray's Inn Road. Assistant Surgeon. Also Third Assistant Anæsthetist.
Derby, Derbyshire Royal Infirmary.—Assistant House Surgeon.
Egyptian Government, Ministry of Education.—Professor of Midwifery and Gynæcology. Also Medical Tutor and Registrar to Kasr-el-Ainy Hospital.
Gloucester General Infirmary and Gloucestershire Eye Institution.—Assistant House Surgeoncy.
Great Northern Central Hospital.—Ophthalmic Surgeon.
Hartlepools Hospital.—House Surgeon.
Hastings, St. Leonards, and East Sussex Hospital.—Assistant House Surgeon.
Hospital for Women, Soho Square, W.—Medical Registrar.
Manchester, Chorlton-upon-Medlock Dispensary.—Resident House Surgeon.
Manchester, University of.—Junior Demonstrator in Physiology.
Norwich, Norfolk and Norwich Hospital.—Surgeon and Assistant Surgeon.
Royal Dental Hospital of London.—Demonstrator in Mechanical Pupils' Department.
Sheffield Royal Hospital.—Assistant House Surgeon.
Wandsworth Union Infirmary, St. John's Hill, near Clapham Junction.—Junior Assistant Medical Officer.

The General Meeting of the Incorporated Institute of Hygiene was held on July 9, when Sir Wm. Broadbent, Bart., was elected President; and Sir Wm. Bennett, Surgeon-General Cleghorn, Sir Alfred Cooper, Mr. Mayo Robson, and Professor Sims-Woodhead were also elected as Vice-Presidents of the Association.

---

### NOTICE TO CORRESPONDENTS.

All MSS., Letters, Books for Review, and other matters intended for the Editor should be addressed The Editor, "The Hospital," The Hospital Buildings, 28 & 29 Southampton Street, Strand, W.C.

The Editor cannot undertake to return rejected MS., even when accompanied by stamped directed envelope.

**Notice to Advertisers and Subscribers.**

All Advertisements, Orders for Copies of The Hospital, and Business Communications should be addressed to **THE MANAGER (Not to the Editor)**, The Hospital, 28 & 29 Southampton Street, Strand London, W.C.

The Cost of Subscription, post free, is as follows:—Per week, 3½d.; per quarter, 3s. 3d.; per half-year, 6s. 6d.; per annum, 13s. Foreign and Colonial:—Per annum, 19s. 6d., payable, in all cases, in advance.

**NOTICE.—Vol. XXXIX. of "The Hospital" (October 1905 to March 1906), in handsome cloth binding, will shortly be on sale at the office of this Journal, price 10s. 6d. Cases for binding the Numbers contained in this Volume 2s. Index to Vol. XXXIX., 3½d., post free.**

**The Monthly Part for July is now ready. Price 1s., by post, 1s. 3d.**

*Just Published.*

**An Important New Work for Students of Anatomy, Masseuses, and Nurses.**

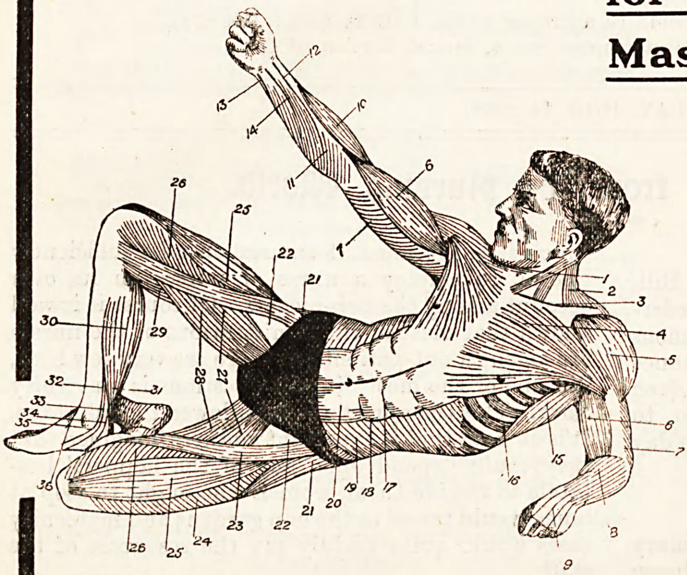

### MUSCLES AND NERVES

**3/6 net, 3/9 post free.**

**An Atlas of the Superficial Muscles and the Principal Motor Nerves of the Human Body for the use of Students of Anatomy and Nurses**

**3/6 net, 3/9 post free.**

By LOUIS B. RAWLING, F.R.C.S.,

*Assistant Surgeon and Senior Demonstrator of Anatomy, St. Bartholomew's Hospital.*

This Atlas contains a series of photographs of a well-developed male in such positions calculated to show off the superficial muscles to the best advantage. Opposite to each figure is a plan which not only illustrates the arrangement and direction of the muscles called into action, but also indicates the situation of the more important motor nerves.

The work cannot fail to be of the greatest practical utility to all Nurses and those who desire to gain a thorough insight into the superficial muscular formation of the human body, and obtain a general idea as to the position of the nerves which supply those muscles.

*Catalogue of Nursing Manuals sent post free on application.*

**The Scientific Press, Ltd.,**

**Publishers of Nursing Text-Books, Charts, and Case-Books of all Descriptions.**

**28 & 29 SOUTHAMPTON STREET, STRAND, LONDON, W.C.**

